# Dataset on microstructural, structural and tribology characterization of TiC thin film on CpTi substrate grown by RF magnetron sputtering

**DOI:** 10.1016/j.dib.2020.105205

**Published:** 2020-01-25

**Authors:** Olayinka Oluwatosin Abegunde, Esther Titilayo Akinlabi, Philip Oluseyi Oladijo

**Affiliations:** a Department of Mechanical Engineering Science, University of Johannesburg, Johannesburg, South Africa; b Department of Chemical, Materials and Metallurgy Engineering, Botswana Institute of Science and Technology, Palapye, Botswana

**Keywords:** RF magnetron sputtering, TiC thin film, Field emission scanning electron microscope (FESEM), Atomic force microscope (AFM), Grazing incidence X-ray diffractometer (GIXRD), Raman spectroscopy, Nanohardness

## Abstract

The datasets in this article are supplementary to the corresponding research article [1, 2]. The planar morphology and topography of TiC thin films coated on commercially pure Titanium (CpTi) grown by RF magnetron sputtering were investigated using Field emission scanning electron microscope (FESEM) and Atomic force microscope (AFM). The mechanical properties such as Hardness and Young Modulus of the thin film coating was studied using Nanohardness. Furthermore, grazing incidence X-ray diffractometer (GIXRD) and Raman spectroscopy were used to analyse the structural and composition of the TiC thin film coating.

**Table:** Specification Table

Subject	Surfaces, Coatings and Films
Specific subject area	RF Magnetron sputtering coating and nanomaterial thin film characterizations.
Type of data	Table Image Figure
How data were acquired	• ZEISS Gemini*2 Field emission scanning electron microscope (FESEM), IIT, Kharagpur, India. • Hysitron Triboindenter T1950 Nanohardness, IIT Kharagpur, India • VEECO Atomic force microscope (AFM) Wits University, South Africa • PANalyticals's Xpert Pro Grazing incidence X-ray diffractometer (GIXRD), India. • Alpha 300R WITEC Raman spectroscopy, University of Johannesburg, South Africa.
Data format	Raw Analysed
Parameters for data collection	The CpTi substrates were polished and ground using ASTM standard. Further cleansing in acetone, isopropanol and deionized water were performed Pre-sputtering for 5 mins to remove contaminants Samples characterizations were conducted in ambient condition
Description of data collected	The FESEM images were captured on the microscope using the ZEISS software at a magnification of X50000 The AFM images of the height profile were captured using nanoscope software The indentation depth of the nanohardness was 10% of the film thickness and the load control mode was used. The Raman was conducted in ambient conduction at an integration time of 10 seconds. The GIXRD was done at an angle of 0.02° from 10° to 90°
Data source location	India Institute of Technology, Kharagpur, India University of Witwatersrand, Johannesburg, South Africa University of Johannesburg, Johannesburg, South Africa
Data accessibility	Data are available on a public repository Repository name: Mendeley Data https://doi.org/10.17632/c25mgp6ptz.1 URL:https://data.mendeley.com/datasets/c25mgp6ptz/1
Related research article	O.O. Abegunde, E.T Akinlabi and O.P Oladijo, “GIXRD, Raman and Surface analysis of TiC thin film coating produced by RF magnetron sputtering,” in Journal of Physics: Conference Series, 2019. O.O. Abegunde, E.T Akinlabi and O.P Oladijo and J. D. Majumdar, “Surface Integrity of TiC Thin Film Produced by RF Magnetron Sputtering,” Procedia Manufacturing, 2019.

**Table:** 

**Value of Data** • The data provide an insight into the significance and influence of thin film coatings on properties of metals • These data can be used for research applications and industrial usage in the area of surface coatings, materials application and mechanical engineering • The data is applicable for the development of predictive and mathematical models for optimization of RF process parameters • The data presents the effect of RF magnetron sputtering process parameters on the coating properties • The data can be explored by scientists and researchers in the field of materials and mechanical engineering

## 1 Data description

**Fig. 1:**
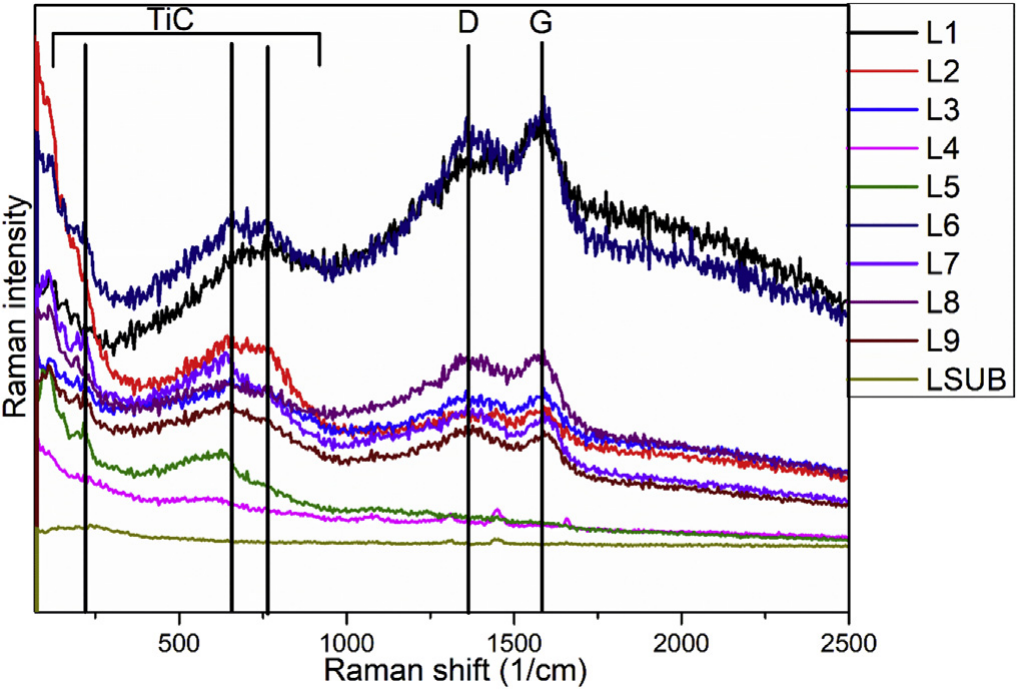
Raman Spectra of TiC thin film coatings.

**Fig. 2:**
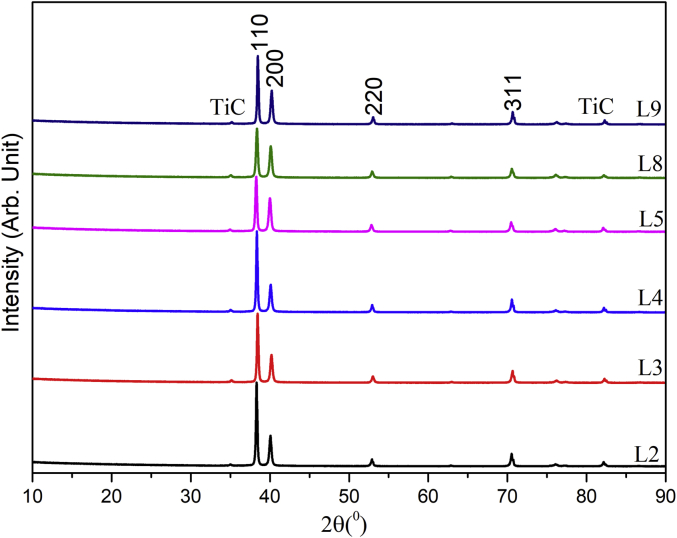
GIXRD diffractogram of the TiC thin film coating.

**Fig. 3:**
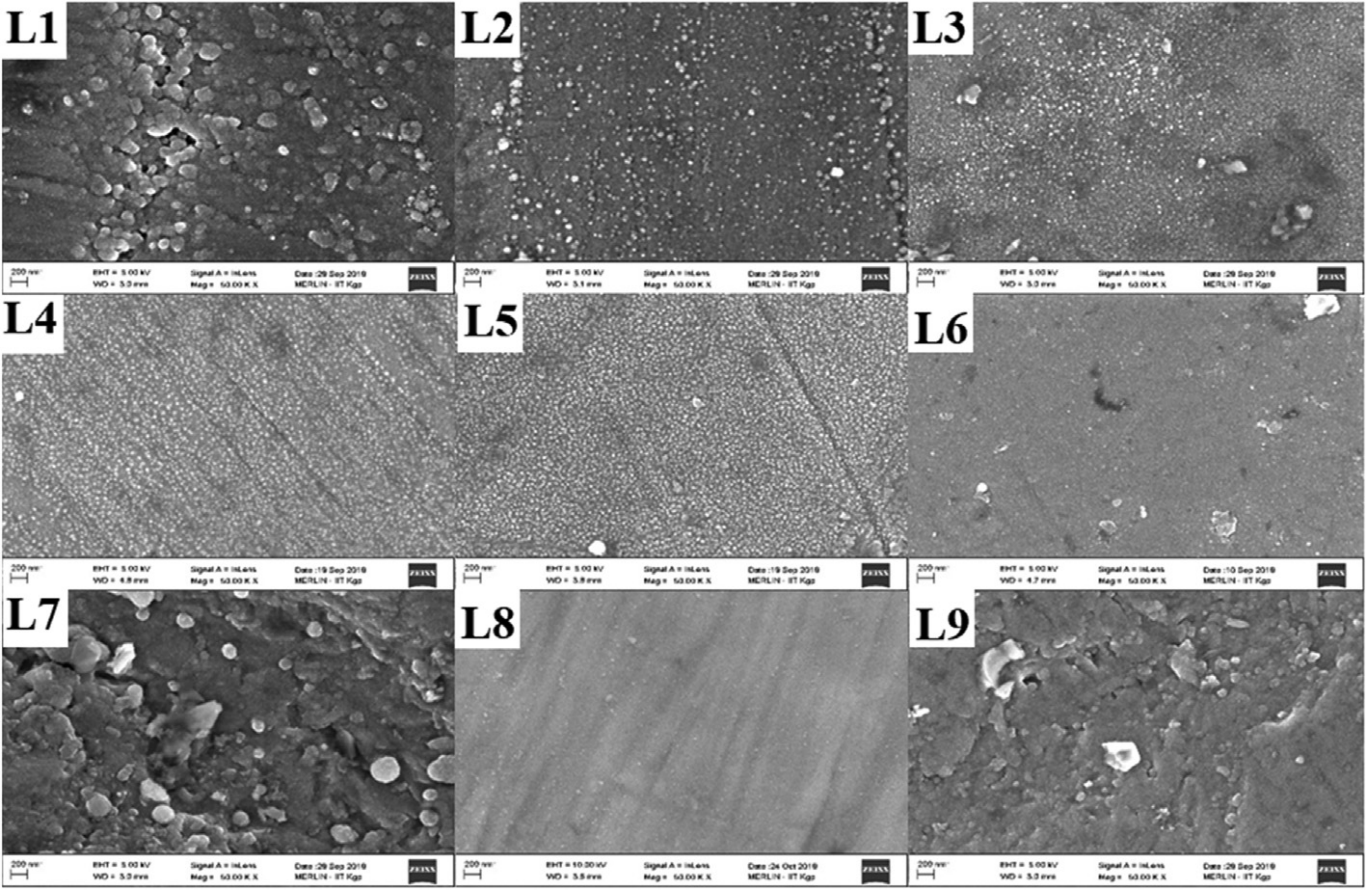
Planar view of the microstructural morphology.

**Fig. 4:**
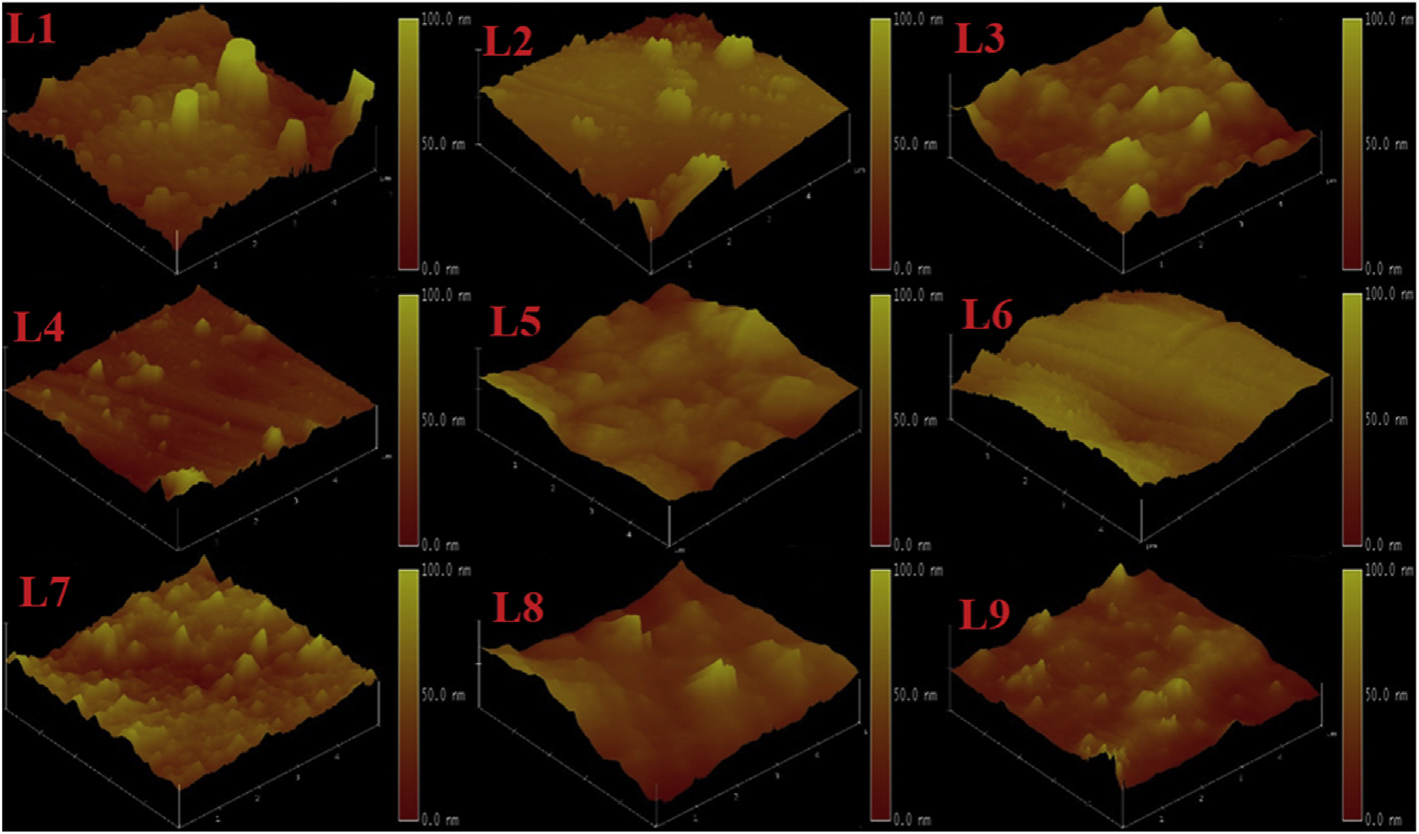
3D view of the surface topography.

**Fig. 5:**
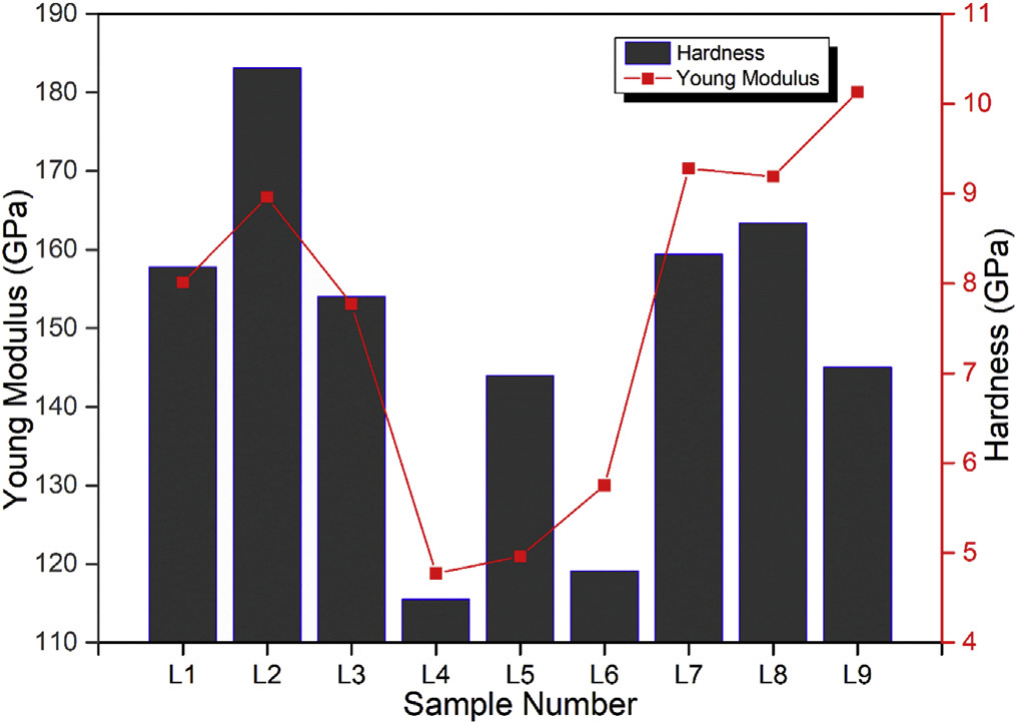
Plot of Sample numbers against the Young Modulus and Hardness.

The RF magnetron sputtering process parameters develop by using L9 Taguchi orthogonal array is presented in Table 1. The Raman spectra of the TiC thin film coatings obtained from Raman spectroscopy is presented in Fig. 1. The GIXRD diffractogram of the structural properties is shown in Fig. 2 and the GIXRD parameters of the TiC thin film coatings such as crystalline size, dislocation density, microstrain and texture coefficient are tabulated in Table 2. Fig. 3 illustrates the planar microstructural morphology evolution of the TiC thin film coating obtained from FESEM analysis while the surface topography result from the AFM analysis is presented in Fig. 4. The AFM statistical information about the surface topography such as mean roughness, skewness and kurtosis are tabulated in Fig. 4 (see Table 3). The output response and plots of the load against displacement are tabulated in Table 4. The plot of the sample numbers against the young modulus and hardness is presented in Fig. 5.

**Table 1:** Experimental matrix for deposition.

S/N	L1	L2	L3	L4	L5	L6	L7	L8	L9
Power (W)	150	150	150	200	200	200	250	250	250
Time (Hrs)	2.0	2.5	3.0	2.0	2.5	3.0	2.0	2.5	3.0
Temperature (^o^C)	80	90	100	90	100	80	100	80	90

**Table 2:** GIXRD output data for CpTi/TiC thin film Coatings.

Experimental run	2Ɵ (^0^)	d spacing (Å)	D ASTM (Å)	FWHM (^0^)	(hkl)	Crystalline size (nm)	Dislocation density (δ) (Lines/m^2^) × 10^14^	Micro strain (ε) × 10^−4^	Texture coefficient
L2	38.3091	2.3476	2.499	0.1092	110	77.0	1.6860	4.5010	8.67
	40.0757	2.2481	2.1637	0.2184	200	38.72	6.6705	8.9526	2.40
	52.9020	1.7293	1.5302	0.1404	220	63.20	2.5037	5.4848	0.90
	70.5517	1.3338	1.3047	0.0936	311	103.96	0.9252	3.33412	3.40
L3	38.4336	2.3403	2.499	0.1404	110	59.92	2.7850	5.7848	8.31
	40.2095	2.2410	2.1637	0.1872	200	45.19	4.8966	7.6704	2.56
	53.0352	1.7253	1.5302	0.156	220	56.91	3.0874	6.0907	0.98
	70.9740	1.3318	1.3047	0.1092	311	89.29	1.2543	3.8821	3.65
L4	38.3424	2.3457	2.499	0.1248	110	67.39	2.2017	5.1434	8.73
	40.1102	2.2463	2.1637	0.234	200	36.14	7.6558	9.5911	2.23
	52.9374	1.7283	1.5302	0.1092	220	81.27	1.5141	4.2653	1.00
	70.5817	1.3333	1.3047	0.1092	311	89.13	1.2588	3.8891	3.58
L5	38.2852	2.3510	2.499	0.1919	110	43.82	5.2075	7.9102	7.17
	39.9825	2.255	2.1637	0.255	200	33.05	9.1561	10.4889	3.44
	52.823	1.733	1.5302	0.102	220	86.71	1.33011	3.9978	1.15
	70.484	1.334	1.3047	0.171	311	56.68	3.1122	6.1152	3.31
L8	38.3539	2.3469	2.499	0.2175	110	38.67	6.6868	8.9636	7.10
	40.075	2.245	2.1637	0.24	200	34.80	8.2578	9.9610	3.49
	52.9069	1.7306	1.5302	0.1023	220	86.74	1.3292	3.9963	1.17
	70.5457	1.3339	1.3047	0.156	311	62.38	2.5701	5.5571	3.31
L9	38.4632	2.3385	2.499	0.1404	110	59.93	2.7845	5.7842	7.80
	40.2185	2.2404	2.1637	0.2184	200	38.74	6.6644	8.9486	2.97
	53.0506	1.7248	1.5302	0.1092	220	81.31	1.5126	4.2632	1.03
	70.6954	1.3314	1.3047	0.1248	311	78.04	1.6419	4.4416	3.50

**Table 3:** AFM images of the thin film taken at 5 × 5 μm^2^ with Statistical detail.

S/N	Z-range (nm)	RMS (nm)	Mean Roughness (nm)	Skewness	Kurtosis
L1	252	28.830	18.34	0.41	3.342
L2	397.25	51.593	39.684	0.263	3.898
L3	320.68	47.506	36.906	0.611	3.807
L4	268.35	30.579	20.482	0.646	4.316
L5	823.89	34.56	24.734	0.536	4.081
L6	537.62	30.72	25.632	0.159	2.307
L7	194.85	57.197	41.392	0.09	3.110
L8	44.66	53.467	40.330	0.451	4.223
L9	232.99	57.837	41.570	0.970	4.408

**Table 4:** Hardness and Young Modulus at different Process Parameters.

S/N	The plot of Load against displacement	Nanohardness Statistical Parameters
L1		Young Modulus (GPa) – 157.81 Hardness (GPa) – 8.01 Wear resistance – 0.02 Plasticity index – 0.051 H_max_ – 28.32 H_F_ – 11.73 % Recovery – 58.58 Plasticity – 41.42
L2		Young Modulus (GPa) – 183.12 Hardness (GPa) – 8.96 Wear resistance – 0.02 Plasticity index – 0.049 H_max_ – 25.99 H_F_ – 11.09 % Recovery – 57.34 Plasticity – 42.66
L3		Young Modulus (GPa) – 154.06 Hardness (GPa) – 7.77 Wear resistance – 0.02 Plasticity index – 0.050 H_max_ – 28.94 H_F_ – 13.81 % Recovery – 52.27 Plasticity – 47.73
L4		Young Modulus (GPa) – 115.55 Hardness (GPa) – 4.77 Wear resistance – 0.0081 Plasticity index – 0.041 H_max_ – 37.89 H_F_ – 15.20 % Recovery – 59.89 Plasticity – 40.11
L5		Young Modulus (GPa) – 143.99 Hardness (GPa) – 4.96 Wear resistance – 0.0059 Plasticity index – 0.034 H_max_ – 35.62 H_F_ – 21.26 % Recovery – 40.33 Plasticity – 59.67
L6		Young Modulus (GPa) – 119.11 Hardness (GPa) – 5.75 Wear resistance – 0.013 Plasticity index – 0.048 H_max_ – 35.07 H_F_ – 13.03 % Recovery – 62.83 Plasticity – 37.17
L7		Young Modulus (GPa) – 159.46 Hardness (GPa) – 9.28 Wear resistance – 0.031 Plasticity index – 0.058 H_max_ – 26.38 H_F_ – 10.86 % Recovery – 58.82 Plasticity – 41.18
L8		Young Modulus (GPa) – 163.41 Hardness (GPa) – 9.19 Wear resistance – 0.030 Plasticity index – 0.056 H_max_ – 29.70 H_F_ – 13.98 % Recovery – 52.95 Plasticity – 47.05
L9		Young Modulus (GPa) – 145.08 Hardness (GPa) – 10.13 Wear resistance – 0.050 Plasticity index – 0.070 H_max_ – 26.91 H_F_ -7.96 % Recovery – 70.41 Plasticity – 29.59

## 2 Experimental design, materials and methods

### 2.1 Deposition of TiC thin film on CpTi using RF magnetron sputtering

TiC thin films were deposited in an RF Magnetron sputtering on CpTi substrate under different process parameters. The purity of the stoichiometric TiC target used is 99.99% pure TiC. The process parameters varied for the deposition processes were deposition time, power and temperature. An L9 (3^3^) experimental array shown in Table 1 was used with three factors at three levels of Low, Mid and High and a total of nine deposition runs were done. Detailed analysis of the deposition condition can be found in Refs. [1,2].

### 2.2 Microstructural and topography characterization

Atomic force microscopy (Veeco Di2100 AFM) was used to evaluate the 3D surface topographies of the samples. Image scan size of 5 × 5 μm^2^ was obtained in tapping mode. All analysis was performed in ambient temperature. Nanoscope software was used for capturing and analyzing the images from the surface of the samples. Field Emission Scanning Electron Microscope FESEM (ZEISS Gemini*2, Germany) capable of capturing nanoscale images effortlessly at very high magnification was used to observe the surface morphology evolution. The FESEM can take images at very high magnification and images were taken at 50,000x magnification.

### 2.3 Structural and composition characterization

Raman analyses were carried out on TiC thin films using an alpha300R (WITec) confocal laser Raman microscope configured with a frequency-doubled Nd-YAG laser (wavelength 532 nm). Raman spectra were collected using ×50 Nikon objectives. A laser power of 2 mW at room temperature was used to prevent burning of the film surface. Beam centring and Raman spectra calibration were performed before spectral acquisition using a-Si standard (111). The Raman spectrum of the substrate was obtained and used to compare with the TiC thin films deposited. Grazing Incidence X-ray Diffractometer (GIXRD PANalyticals's Xpert Pro with Cu K-alpha and wavelength 1.540598 A) at a very low angle of incidence of 0.02°/s from 10° to 90° was used to study the structural properties of the thin film. The crystallite size (D) was calculated using the Scherer equation, D= (0.9 λ)/βcosθ; where λ is the wavelength of the X-ray used (1.540598 A); β, the full width at half maximum (FWHM) of the highest-intense peak; and θ, the Bragg angle.

### 2.4 Mechanical characterization

Nanomechanical properties such as Young's modulus and hardness of thin films were obtained by nanoindentation technique (Hysitron, Triboindenter TI950, USA). Load controlled indentation testing followed a trapezoidal (loading-dwelling-unloading) profile with a hold time of typically 15 s at peak load. The peak load was 300μN at a loading rate of 10 μN s^−1^. The diamond indenter was a Berkovich tip with a tip radius of curvature of 100 nm. From the analyzed load-displacement curves, Young's modulus of measured films can be calculated using Oliver and Pharr analysis [3,4]. All the data presented in this study corresponds to an average of 10 measurements. The indentation depth was never deeper than 10% of the total coating thickness to avoid the influence of the substrate on the coating [5].
